# Unexpected Increase in Bone Mineral Density With Rapamycin and Low-Dose Naltrexone: A Case Report of a 52-Year-Old Woman With Osteopenia

**DOI:** 10.7759/cureus.77435

**Published:** 2025-01-14

**Authors:** Amy Britton, Girish Harinath, Stefanie Morgan, Sajad Zalzala

**Affiliations:** 1 Longevity Medicine, AgelessRx, Ann Arbor, USA; 2 Geroscience, AgelessRx, Ann Arbor, USA

**Keywords:** aging women, bone density, gerotherapeutics, healthy aging, longevity medicine

## Abstract

Osteopenia and osteoporosis are prevalent bone disorders characterized by reduced bone mineral density (BMD), leading to an increased risk of fractures. This case report presents a 52-year-old Caucasian female patient with osteopenia who experienced an unexpected 15.9% increase in lumbar spine BMD within two years after enrolling in a clinical trial involving low-dose rapamycin and subsequently starting low-dose naltrexone. This case potentially opens novel treatment strategies for bone density improvement in aging populations.

## Introduction

Osteoporosis is a skeletal disease characterized by decreased bone mineral density (BMD) and altered structure, which compromises bone strength, predisposing individuals to an increased risk of fractures [1]. Osteopenia is a condition that precedes osteoporosis and represents an earlier stage of the degenerative pathology. Osteopenia is diagnosed when BMD T-scores fall between -1.0 and -2.5 standard deviations below the mean of a young adult reference population, while osteoporosis is defined by T-scores of -2.5 or lower [2]. The World Health Organization estimates that over 200 million people worldwide are affected by osteoporosis, with postmenopausal women being particularly susceptible due to the rapid decline in estrogen levels. Estrogen plays a crucial role in regulating bone homeostasis through its multi-faceted effects on cell signaling within the bone microenvironment, stimulating osteoblast (bone-mineralizing) and inhibiting osteoclast (bone resorptive) activity [3,4]. Other drivers of osteoporosis pathology include vitamin D and calcium deficiencies, chronic inflammation, oxidative stress, cellular senescence, and other hormone imbalances such as testosterone and cortisol [5].

BMD serves as a key indicator of bone strength and fracture risk, reflecting the complex interplay between bone formation and resorption [6]. This dynamic process, known as bone remodeling, is regulated by various factors, including hormones, cytokines, and an array of metabolites and mechanical stimuli [7]. During the biological aging process, the balance shifts toward increased bone resorption relative to formation, leading to a net loss of bone mass and deterioration of bone microarchitecture that leads to the development of osteopenia and osteoporosis [8]. Further, various lifestyle factors (e.g., smoking, sedentary behavior, inadequate micronutrient consumption), menopause, certain medications, and toxin exposure further aggravate this imbalance and accelerate disease progression [9,10]. The relationship between BMD and fracture risk is inverse and exponential, with each standard deviation decrease in BMD associated with a 1.5 to 3-fold increase in fracture risk [11-13]. Vertebral and hip fractures are among the most prevalent and have a high mortality rate in individuals over 60, with those that survive often losing functional independence and having a severely compromised quality of life [14,15]. Consequently, accurate measurement and early monitoring of BMD through biomarkers of bone breakdown (e.g., C-telopeptide cross-linked type I collagen) and techniques such as dual-energy X-ray absorptiometry (DXA) are essential for diagnosis, risk assessment, and evaluating the clinical effectiveness of preventative regimens [16,17].

Standard treatment options for osteopenia and osteoporosis aim to prevent fractures by increasing or maintaining BMD and improving bone quality [18]. First-line pharmacological interventions typically include antiresorptive agents such as bisphosphonates, which inhibit osteoclast activity, and selective estrogen receptor modulators (SERMs) [19]. For more severe cases or when antiresorptive therapies are contraindicated, anabolic agents like teriparatide or abaloparatide may be employed to stimulate bone formation [20]. While very effective for some, hormone replacement therapy faces rigorous regulatory barriers and has demonstrated varying efficacy across age groups and based on individual patient risk factors [21,22]. Supplementation with calcium and vitamin D, along with lifestyle modifications including weight-bearing exercise and smoking cessation, complement pharmacological approaches [23]. Collectively, these therapies typically result in BMD increases ranging from 2% to 10% over 1-3 years, depending on the specific treatment and skeletal site [24].

Despite these interventions, significant challenges persist in the prevention and management of osteopenia and osteoporosis. These include suboptimal medication adherence, concerns about rare but serious side effects such as atypical femoral fractures and osteonecrosis of the jaw with long-term bisphosphonate use, and the limited duration of safe and effective use for anabolic agents [25,26]. Furthermore, a substantial proportion of patients fail to achieve significant improvements in BMD with standard treatment protocols, underscoring the need for novel treatment strategies and personalized, preventative approaches to optimize bone health across diverse patient populations [27]. Geroprotective interventions target fundamental cellular and molecular mechanisms that mediate the biological aging process to ameliorate drivers of age-related decline in multiple tissues and enhance healthy longevity [28]. Rapamycin and low-dose naltrexone are promising geroprotectors demonstrated to mitigate or prevent several age-related diseases and extend lifespan in model organisms. Though rapamycin and LDN are approved for organ rejection and opioid dependence, respectively, they have been demonstrated to exert their geroprotective effects at lower doses. Both LDN and rapamycin target and remedy molecular mediators of low-level, sterile inflammation that is a driver of bone resorption and degeneration, as well as enhance osteoblast activity through modulation of opioid receptor signaling and mTOR activity, respectively [29-34]. In this report, we describe a case of significant improvement of 15.9% in BMD over two years with a novel therapeutic approach that includes putative geroprotective interventions in a 52-year-old female diagnosed with osteopenia. This suggests further exploration of this therapeutic strategy may be beneficial in a broader population of post-menopausal women for bone density improvement.

## Case presentation

A 52-year-old Caucasian female presented with a history of osteopenia, initially diagnosed in 2019 at age 47. At the time of her initial diagnosis, a DXA scan revealed a T-score of -1.0 at the lumbar spine and -1.3 at the femoral neck. In response to these findings, a comprehensive treatment plan was initiated in 2019, which consisted of hormone replacement therapy, including transdermal estradiol administered as a 0.1% gel at a dose of 1 mg/day and a compounded testosterone cream (4 mg/g) applied at a variable dose of 1-4 mg/day. These interventions were in addition to a Mirena intrauterine device (IUD) placed in 2018 to provide endometrial protection.

**Figure 1:**
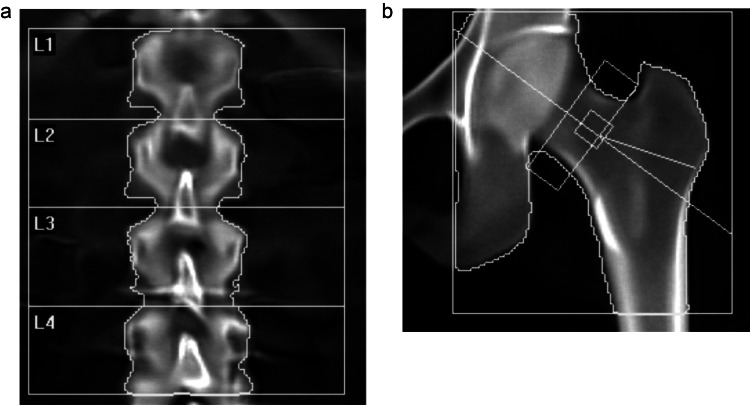
Bone mineral density after conventional intervention from November 2019 to October 2021 Dual-energy X-ray absorptiometry (DXA) scans showed no change in the baseline lumbar spine T-score of -1.0 (a) and only a non-significant improvement in the femoral neck T-score to -1.2 (1.9% improvement, within the error range of the machine) from -1.3 (b) after two years of conventional interventions.

Concurrent with the pharmacological interventions, the patient received lifestyle recommendations to support bone health. These included engaging in mixed-impact weight-bearing cardiovascular exercise for a minimum of 150 minutes per week, participating in resistance training sessions twice weekly, supplementing with vitamin D3, and maintaining a dietary calcium intake of 650-720 mg per day through the consumption of 5-7 servings/week of cottage cheese, salmon, sardines, and Greek yogurt. Despite these collective interventions, a follow-up DXA scan conducted after two years at age 49 in 2021 showed virtually no change in BMD at the lumbar spine (Figure 1a) and no meaningful femoral neck measurement improvement (T-score of -1.2, 1.9% improvement that was within the expected coefficient of variation for the DXA scanner; Figure 1b), indicating that the initial treatment approach had effectively stabilized the patient's bone density but had not led to significant improvements.

In April 2022, at age 50, the patient enrolled in a double-blinded, placebo-controlled trial investigating the use of low-dose rapamycin for longevity and health span enhancement (PEARL, NCT04488601 [35]). The primary goal of the study was to validate the safety of 12 months of weekly, low-dose rapamycin administration and assess initial efficacy signals for improvements in body composition, quality of life, and an exploratory array of biomarkers of aging. Concurrent with this, beginning in January 2023, the patient also independently began supplementing with relatively low doses of vitamin K2 (MK4, 500-700 mcg) daily and incorporated regular consumption of natto, a fermented soybean product rich in vitamin K2, into her diet. Her trial participation was completed in April of 2023, and she continued taking low-dose commercial rapamycin (titrated from 2 mg up to 6 mg) thereafter. In July 2023, the patient's treatment regimen was further modified with the introduction of low-dose naltrexone at an initial dose of 1.5 mg taken nightly, which was progressively increased to 4.5 mg nightly over the course of several weeks. Later that year, in November 2023, she commenced oral progesterone supplementation at a dose of 100 mg nightly (interventional timeline in Table 1). Following the PEARL trial unblinding in early 2024, it was revealed that the patient had been in the active arm, receiving 5 mg of compounded rapamycin once weekly (though subsequent studies suggested that due to lesser availability of compounded rapamycin relative to commercial formulations, this was equivalent to approximately 1.67 mg).

**Table 1:** Intervention and DXA scan assessment timeline DXA: dual-energy X-ray absorptiometry; all interventions were consistently engaged throughout the length of the trial *15.9% change relative to baseline

				DXA				
Date	Age	BMI	Ethnicity	Lumbar spine T-score	Femoral neck T-score	Total body T-score	Interventions	Relevant symptoms to bone health
2018	46	22.9	Caucasian				Mirena intrauterine device	Intermittent low back pain
Baseline (Nov 2019)	47	21.5		-1	-1.3		None	
Nov 19	47						Hormone Replacement Therapy (transdermal estradiol as 0.1% gel at 1 mg/day, compounded testosterone cream (4 mg/g) applied at a variable dose of 1-4 mg/day); vitamin D3, dietary calcium intake of 650-720 mg per day (5-7 servings/week of canned sardines, salmon, cottage cheese, Greek yogurt); mixed impact weight-bearing cardiovascular exercise for a minimum of 150 minutes per week, participating in resistance training sessions twice weekly	
Oct 21	49	22.8		-1	-1.2		No change	
May 22	50	23.7				0.6	Rapamycin (5 mg compounded)	
Jan 23	51	24.1					Vitamin K2 at 500-700 mcg daily, natto	
May 23	51	24.9					Switch to Rapamycin 2-6 mg generic	
Jul 23	51	25					Low dose naltrexone (1.5 --> 4.5 mg)	
Nov 23	51	25					Oral progesterone (100 mg) nightly	
Apr 24	52	23.3		0.3*	-1.1	0.7	No Change	

**Figure 2:**
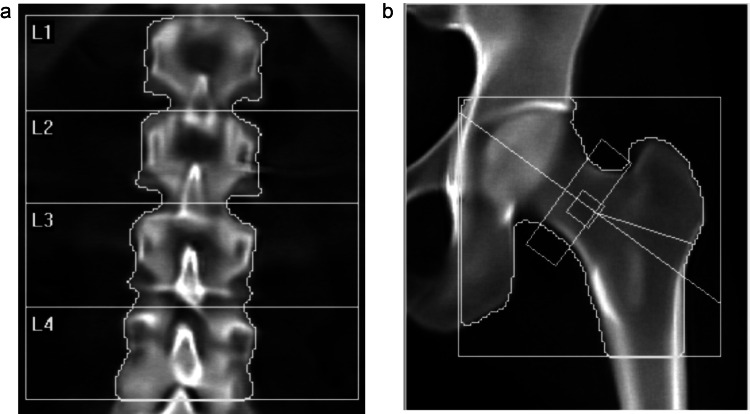
Bone mineral density after novel combinatorial interventions from May 2022 to April 2024 Following two years of using rapamycin, with periodic additions of nutraceuticals (vitamin K2), hormones (oral progesterone), and a geroprotector (low-dose naltrexone), DXA scans showed a 15.9% improvement in baseline lumbar spine T-score of 0.3 (a) and a slight, non-significant improvement in the femoral neck T-score to -1.1 (3.9% improvement) from -1.3 (b).

DXA scans in April 2024 (performed at the same facility as previous scans) after the PEARL trial conclusion yielded remarkably improved results. Specifically, the scan revealed a 15.9% increase in lumbar spine BMD (to T-score of 0.3; Figure 2a) over the two years that elapsed since beginning this novel regimen for treating her osteopenia, though the femoral neck BMD showed only a non-significant increase (to T-score of -1.1, or 3.9%; Figure 2b). This is consistent with non-diagnostic whole-body BMD DXA measurements obtained throughout the PEARL trial, showing improvements from a T-score of 0.5 at the start of the trial to 0.7 at the end (Table 1). These findings represent an unexpected and substantial improvement in the patient's bone health, which was particularly notable given the typical challenges in achieving significant BMD increases in patients with osteopenia, especially post-menopausal women.

## Discussion

The substantial increase in lumbar spine BMD observed in this case study following novel interventions is noteworthy, rivaling improvements seen with FDA-approved osteoporosis medications [36]. These are perhaps all the more impactful considering the unexpected nature of this finding - the patient began taking low-dose rapamycin and low-dose naltrexone to minimize general age-related decline, not in an effort to improve BMD specifically. While it is important to note that the patient’s additional dietary, lifestyle, and supplement changes were engaged with throughout the length of the trial and may have played a role, the collective impacts of these interventions are significant.

Several mechanisms may have contributed to the observed increase in BMD. For example, rapamycin's effects on bone health could be mediated through its regulation of the mammalian target of rapamycin (mTOR) pathway, which plays a crucial role in bone remodeling by modulating osteoclast activity and enhancing autophagy in bone cells to promote a more favorable balance between bone formation and resorption [37,38]. Additionally, low-dose naltrexone may influence bone health through multiple pathways, such as modulating immune activity and blunting chronic inflammation to benefit bone signaling and metabolism, or through improvements in bone health associated with opioid receptor antagonism [29-32]. One interesting observation is the substantial lumbar region-specific improvement in BMD compared to the much smaller effect in femoral neck BMD. This may be due to the fact that the lumbar vertebrae have a higher proportion of trabecular (spongy) bone, which is more metabolically active and, in several cases, has been shown to respond relatively quickly to anti-inflammatory interventions (e.g., TNF-alpha). Further, the lumbar spine experiences greater mechanical loading, stimulating bone remodeling. Anti-inflammatory medications that reduce inflammation-related bone degradation may synergize with resistance training to stimulate a more pronounced improvement in BMD in the lumbar spine [39,40].

While further investigation as to which of the interventions or combinations of interventions described here most reliably improves BMD in post-menopausal women is required, the improvements in BMD in this patient surpass what has previously been reported for any of these therapies independently, as well as what is observed with most conventional treatment approaches. Though the impacts of low-dose rapamycin are not extensively characterized, improvements in BMD in this magnitude have not yet been reported for this therapy alone. Similarly, while naltrexone has been suggested to improve BMD for some users, improvements of this magnitude have not previously been observed. We hypothesize that synergistic effects of low-dose rapamycin and low-dose naltrexone, in conjunction with diet, supplements, and lifestyle changes, were a significant driver of the magnitude of improvement seen in this patient. Improvements in femoral neck BMD from baseline were small (3.9%) and likely not clinically significant, but they fell outside the expected margin of error of DXA scanners (1-2%) utilized in the study and the standard expected variability of femoral neck BMD (2-2.5%) as measured by DXA scanners and published in prior literature [41].

The findings of this case study highlight the potential impact of combinatorial intervention strategies (including noncanonical therapies) for eliciting substantial improvements in aging biology and mitigating age-related decline. Despite this promise, there are several limitations to the study. While this case study allowed more in-depth and comprehensive characterization of the individual's health journey over a longitudinal timeframe, validation of this in a larger population is required before such conclusions can be decisively drawn. Further, the participant engaged with several intervention modalities at different time points, dosages, formulations, and regimens. As K2 promotes osteocalcin carboxylation and enhances calcium binding to the bone matrix, it likely contributes further to bone mineralization [42,43]. Even though the doses of vitamin K2 utilized in this study were low, contributions of vitamin K2, with or without other therapies, should not be overlooked. Rigorously controlled studies, including standardized protocols and measures, will be important for further clarity on whether the combinatorial regimen of rapamycin and LDN substantially improves BMD. Regardless, the potential synergistic interaction between these interventions presents an intriguing avenue for further research in the field of bone health and osteoporosis prevention and treatment.

## Conclusions

This case highlights the potential of a novel combinatorial strategy including putative gerotherapeutic drugs for improving BMD. The remarkable 15.9% increase in lumbar spine BMD over two years suggests that the combination of low-dose rapamycin and low-dose naltrexone, possibly in conjunction with vitamin K2 supplementation, may offer a novel approach to mitigating the consequences of age-related decline in bone health and treating osteopenia and osteoporosis, thereby preventing a major cause of morbidity and mortality in elderly individuals. As this is a single observational case study, further research is needed to elucidate the mechanisms involved and the relative influence of various components of the combinatorial intervention strategy and to determine the efficacy, safety, and generalizability of this approach in larger populations.
